# Effect of local prolonged-release incisional doxycycline on surgical site infection prophylaxis in abdominal colorectal surgery: the SHIELD 1 randomized clinical trial

**DOI:** 10.1097/JS9.0000000000001824

**Published:** 2024-06-13

**Authors:** Oded Zmora, Phillip Fleshner, Philip S. Barie, Lior Segev, George M. Viola, Anthony J. Senagore, Antonino Spinelli, Olga Belotserkovsky, Shmuel Sharoni, Noam Emanuel

**Affiliations:** a Department of Surgery, Shamir Medical Center, Be’er Ya’akov; b Department of General and Oncological Surgery-Surgery C, Sheba Medical Center, Tel Hashomer; c Sackler School of Medicine, Tel Aviv University, Tel Aviv; d PolyPid Ltd., Petach Tikvah, Israel; e Division of Colorectal Surgery, Cedars-Sinai Medical Center, Los Angeles, CA; f Department of Surgery, Weill Cornell Medicine, New York, NY; g Department of Infectious Diseases, Infection Control and Employee Health, The University of Texas MD Anderson Cancer Center, Houston, TX; h Colorectal Surgeon. AJS Innovative Solutions LLC, Holland, MI, USA; i Department of Biomedical Sciences, Humanitas University, Pieve Emanuele; j IRCCS Humanitas Research Hospital, Rozzano, Milan, Italy

**Keywords:** colorectal surgery, doxycycline, prevention, prophylaxis, risk factors, surgical site infection

## Abstract

**Introduction:**

Despite advanced infection control practices including preoperative antibiotic prophylaxis, surgical site infection (SSI) remains a challenge. This study aimed to test whether local administration of a novel prolonged-release doxycycline-polymer-lipid encapsulation matrix (D-PLEX) before wound closure, concomitantly with standard of care (SOC), reduces the incidence of incisional SSI after elective abdominal colorectal surgery.

**Materials and methods:**

This was a phase 3 randomized, controlled, double-blind, multinational study (SHIELD 1) between June 2020 to June 2022. Patients with at least one abdominal incision length greater than 10 cm were randomized 1:1 to the investigational arm (D‐PLEX+SOC) or control (SOC) arm. The primary outcome was a composite of incisional SSI, incisional reintervention, and all-cause mortality.

**Results:**

A total of 974 patients were analyzed, of whom 579 (59.4%) were male. The mean age (±SD) was 64.2±13.0 years. The primary outcome occurred in 9.3% of D-PLEX patients versus 12.1% (SOC) [risk difference estimate (RDE), −2.8%; 95% CI (−6.7%, 1.0%), *P*=0.1520]. In a pre-specified analysis by incision length, a reduction in the primary outcome was observed in the greater than 20 cm subpopulation: 8% (D-PLEX) versus 17.5% (SOC) [RDE, −9.4%; 95% CI (−15.5%, −3.2%), *P*=0.0032]. In the greater than 10 to less than or equal to 20 cm subgroup, no reduction was observed: 9.9% versus 7.9% [RDE, 2.0%; 95% CI (−2.8%, 6.7%), *P*=0.4133]. Exploratory post hoc analyses of patients with increased SSI risk (≥1 patient-specific comorbidity) indicated a reduction in the incidence of the primary outcome: 9.0% (D-PLEX) versus 13.7% (SOC) [RDE, −4.8%; 95% CI (−9.5%, −0.1%), *P*=0.0472]. The D-PLEX safety profile was good (no difference in treatment-emergent adverse events between the groups).

**Conclusions:**

The SHIELD 1 study did not meet its primary outcome of reduced incisional SSI, incisional reinterventions, or all-cause mortality. Pre-specified and post hoc analyses suggested that D-PLEX may reduce the incidence of the primary outcome event in patients with increased SSI risk, including lengthy incisions.

## Introduction

Highlights

- SHIELD 1 randomized prospective phase 3 study tested the effect of a local prolonged-release incisional doxycycline (D-PLEX) on surgical site infection (SSI) prophylaxis in abdominal colorectal surgery.
- Overall, the study did not meet its primary outcome of reduced incisional SSI, incisional reinterventions, or all-cause mortality.
- However, in a pre-specified analysis of the subpopulation with lengthy surgical incisions, there was a clinically meaningful reduction in the primary outcome events with D-PLEX.
- Exploratory post hoc analyses of patients with increased SSI risk (procedural or patient-specific SSI risk) also demonstrated a substantial reduction in the primary outcome event.
- D-PLEX efficacy and safety among patients with increased SSI risk is currently being evaluated in an additional Phase 3 clinical trial (SHIELD 2).

Infection control practices have advanced over the past several decades^1–4^; however, surgical site infection (SSI) remains a substantial cause of morbidity, prolonged hospitalization, and mortality^5–9^. Abdominal colorectal procedures are particularly associated with a high SSI rate owing to the inherent risk of bacterial wound contamination^10–15^.

Administration of preoperative antibacterial prophylaxis within 60 min before incision is established as the most consistently effective SSI prevention measure and is globally the standard of care (SOC)^16–18^. SOC systemic antibiotics aim to provide adequate bactericidal serum and tissue concentrations intraoperatively^4,16,19^ so as to protect against microbial contamination but have limited local (incisional) bioavailability after the skin incision is closed. A combination of vasoconstriction, microthrombosis, electrocautery use, and the inflammatory response at the wound site likely leads to wound tissue hypoperfusion and renders prolonged systemic antibiotics ineffective^10^. Attempts to prolong systemic antibiotic exposure after surgical closure via repeated dosing are ineffective and have been associated with increased adverse events^20,21^. An alternative approach may be administering antibiotics locally, just before incision closure in the operating room, which can have prolonged release directly at the surgical site.

Doxycycline-polymer-lipid encapsulation matrix (D-PLEX) is a novel, biodegradable, prolonged-release (30 days) doxycycline delivery system that is applied directly on incisional soft tissue surfaces following fascial closure to prevent SSI^22–25^. D‐PLEX (eFig. 1, Supplement 1, Supplemental Digital Content 1, http://links.lww.com/JS9/C735) was supplied as a sterile powder for reconstitution of the paste. Applied after fascial closure to soft tissues of the abdominal wall, subcutaneous fat, and dermis^24^, D-PLEX supports the continuous release of high local doxycycline concentrations that exceed the minimal inhibitory concentration (MIC) values for common SSI pathogens^23,26^.

In a recent multicenter randomized Phase 2 study in patients undergoing elective abdominal colorectal surgery, D-PLEX was administered concomitantly with SOC systemic antibiotics and demonstrated a 64% risk reduction in SSI incidence compared to SOC alone^24^. D-PLEX was well tolerated without an increase in treatment-emergent adverse events (TEAEs)^24^. Here, we evaluated D-PLEX efficacy and safety in a prospective, multinational, randomized, double-blind, controlled Phase 3 study (SHIELD 1) in patients undergoing elective abdominal colorectal surgery, hypothesizing a reduction of SSI.

## Methods

### Trial design

The SHIELD 1 study assessed the efficacy and safety of D-PLEX administered concomitantly with SOC for the prevention of SSIs in patients undergoing open elective abdominal colorectal surgery (clean-contaminated wound classification). Patients were randomized 1:1 to the investigational arm (D‐PLEX+SOC) or control (SOC) arm. The protocol was approved by the U.S. Food and Drug Administration under an investigational new drug application, and reviewed and approved by each country’s Ministry of Health and the relevant Institutional Review Board or Ethics Committee at each participating site before study initiation. We followed the principles of Good Clinical Practice, the Declaration of Helsinki, the International Council of Harmonization guidelines, and local regulations. All the participants provided written informed consent. This study work has been reported in line with Consolidated Standards of Reporting Trials (CONSORT, Supplemental Digital Content 2, http://links.lww.com/JS9/C736) Guidelines^27^.

### Patients

The eligible patient population included male and female adults (≥18 years) scheduled to undergo open elective abdominal colorectal surgery involving resection with or without stoma creation, with at least one abdominal incision length of greater than 10 cm (target incision). SOC intravenous (IV) antibiotic prophylaxis (first- or second-generation cephalosporin and metronidazole administered 60 min preoperatively) was based on international guidelines and standardized for all study sites. Mechanical bowel preparation (MBP) was performed at the discretion of the surgeon; however, oral antibiotic bowel preparation (OABP) was not allowed to avoid potential confounding. Patients were excluded if they had any preoperative infection in the week before randomization; underwent concomitant surgical procedures via the target incision; received neoadjuvant abdominopelvic radiation or systemic chemotherapy within 4 weeks; received doxycycline or another tetracycline family antibiotic within 4 weeks; had known hypersensitivity to tetracyclines or D-PLEX excipients; or had allergies to more than three substances as determined from the screening questionnaire. At enrollment, patient demographics and medical history were documented, including a Charlson comorbidity index questionnaire^28,29^.

### Study treatment

D‐PLEX (PolyPid Ltd.) consists of a polymer-lipid-based matrix coated on beta-tricalcium phosphate particles^23,26^ that encapsulates doxycycline into a protected reservoir. Each 5 g D‐PLEX vial contains 54.6 g doxycycline (1.09% w/w). D-PLEX dosing was performed according to incision length: greater than 10 to less than or equal to 20 cm, two vials; and greater than 20 cm, three vials, for a maximum of 163.8 mg doxycycline. All surgeons were trained in the preparation and application of D-PLEX. No placebo was administered to the SOC arm.

### Randomization

Patients were randomized 1:1 to D-PLEX+SOC (D-PLEX arm) or SOC alone (control arm) through an interactive web-based system integrated with an electronic case report form. Randomization was stratified by the type of SOC prophylaxis (IV antibiotic with or without MBP) and region (USA, Europe, and Israel) using specifications generated by an independent statistician. Instructions and training for randomization were provided during the site initiation visit.

### Blinding and SSI assessment

The sponsor, wound assessors, and the independent adjudication committee were blinded to the treatment arm assignments. In addition, all site staff involved in collecting, assessing, and recording the clinical and laboratory data were blinded to the treatment arm assignment. The study site personnel who performed the index operation were trained not to disclose the treatment arm assignment. An assessor evaluated the surgical site on postoperative days 1, 5, 14, and 30 according to the U.S. Centers for Disease Control and Prevention criteria^30^. The assessors’ reports were forwarded to an independent adjudication committee (two infectious diseases specialists and a colorectal surgeon) together with a complete synopsis, including microbiology, hematology, and chemistry test results, and photographs of the incision. In cases of disagreement between the assessments, the adjudication committee prevailed.

### Study outcomes

The primary outcome evaluated over 30 days post-surgery was a composite of incisional SSI (superficial or deep), incisional reinterventions of the index incision due to poor wound healing including dehiscence, or all-cause mortality. Key secondary outcomes included SSI rates and the number of patients with at least one ASEPSIS (**A**dditional treatment, the presence of **S**erous discharge, Erythema, or Purulent exudate, Separation of the deep tissues, Isolation of bacteria, and duration of inpatient **S**tay) score greater than 20 (ASEPSIS allots daily points for the appearance of the incision in the first postoperative week and for the clinical consequences of infection)^31^. Additional secondary efficacy outcomes included rates of superficial SSI, deep SSI, all-cause mortality, times to adjudicated SSI, incisional reintervention, or any other surgical reintervention.

Subgroup analyses pre-specified in the study design were undertaken in patients with target surgical incision lengths greater than 10 to less than or equal to 20 cm versus >20 cm and patients treated with or without MBP. A reduction of ~50% in the clinical outcomes was considered meaningful. Safety analyses included adverse events and all-cause mortality within 60 days of the index surgery. Post hoc analyses were conducted in patients with procedural risk (patients undergoing greater than 20 cm incision)^32–36^ and patient-specific risk factors [i.e. obesity (BMI >30 kg/m^2^ ^27^], diabetes mellitus, hypertension, peripheral vascular disease, and chronic obstructive pulmonary disease/smoking)^37–41^ and in patients who underwent colorectal cancer resections.

### Statistical methods

Sample size calculation was based on powering the study for the primary outcome. The assumed baseline incisional SSI incidence in open elective abdominal colorectal surgery was 16% based on published SSI rates^11–15^, SSI rates at the participating sites, and the observed SSI rate in the Phase 2 multicenter study that assessed the safety and efficacy of D-PLEX administered concomitantly to SOC^24^. No D-PLEX effect was assumed regarding mortality or incisional reintervention rates. A sample size of 882 was estimated to provide 90% power to detect a 50% reduction in incisional SSI rate and thus a significant difference in treatment failure proportions based on χ^2^ analysis. To account for an anticipated 5% lost-to-follow-up rate, the recruitment of a minimum of 950 patients (475 per treatment group) was planned.

Demographic data are presented as the mean ± standard deviation. The Cochran–Mantel–Haenszel test was used to compare treatment failure proportions between groups, and stratified risk differences were determined along with the 95% CI. The two-sided significance level set for the primary outcome was 4.874%. Missing data on day 30 were conservatively imputed as a failure event. The *P* values presented for post hoc analyses were nominal and not adjusted for multiplicity. Analyses were performed using SAS version 9.3 (SAS Institute).

## Results

### Participant flow and recruitment

**Figure 1:**
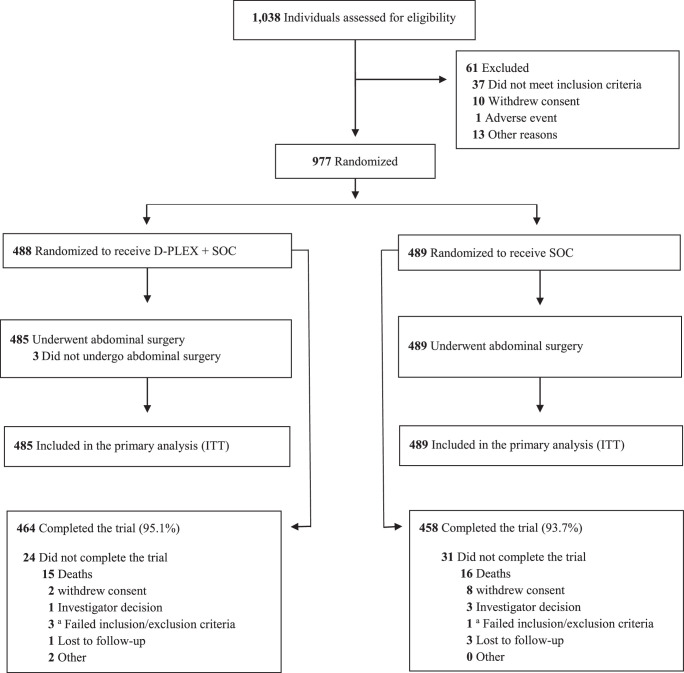
CONSORT diagram. D-PLEX, doxycycline-polymer-lipid encapsulation matrix; SOC, standard of care.

Between 24 June 2020, and 8 June 2022, 1038 patients undergoing open elective abdominal colorectal surgery in 10 countries were screened (Moldova 177, Romania 168, Hungary 160, Israel 140, Czech Republic 132, Bulgaria 92, Croatia 40, United States 37, Poland 17, and Slovakia 14); 977 proceeded to randomization into either the SOC (*n*=489) or D-PLEX arm (*n*=488). In the D-PLEX arm, three patients did not undergo abdominal surgery as randomized (transperineal rectal surgery). The primary outcome analysis was performed using the intention-to-treat (ITT) population, SOC (n=489), and D-PLEX (*n*=485) (Fig. 1).

### Baseline characteristics

There were 59.4% male and 40.6% female patients. The mean age was 64.2±13.0 years, and the mean BMI was 26.96±4.60 kg/m^2^. Overall, the baseline characteristics were similar between the treatment arms, including the Charlson comorbidity index and patient-specific SSI risks. MBP was performed in 84.6% of the patients, with similar rates in both treatment arms. Most patients were diagnosed with cancer (76.5%), followed by benign colorectal disease (19.2%) and inflammatory bowel disease (4.3%). The most common type of surgery performed in both arms was a right colectomy (Table 1).

**Table 1:** Baseline characteristics of study participants (intention-to-treat population).

Characteristics	D-PLEX+SOC (*n*=485)	SOC (*n*=489)
Age (year)	64.7±12.8	63.7±13.3
Sex (male), *n* (%)	288 (59.4)	291 (59.5)
Race, *n* (%)		
White	483 (99.6)	486 (99.4)
Black	1 (0.2)	2 (0.4)
Asian	0	1 (0.2)
Other	1 (0.2)	0
BMI (kg/m^2^)	26.8±4.5	27.1±4.6
CCI Total Score (score range)	4.0 (0–9)	4.0 (0–13)
Indication for abdominal index operation, *n* (%)		
Cancer	369 (76.1)	376 (76.9)
Inflammatory bowel diseases	18 (3.7)	24 (4.9)
^a^Other benign colorectal disease	98 (20.2)	89 (18.2)
Patient-specific SSI risks, *n* (%)		
Diabetes mellitus	88 (18.1)	77 (15.7)
COPD/smoking	79 (16.3)	94 (19.2)
Obesity (BMI ≥30 kg/m^2^)	25 (5.2)	20 (4.1)
Hypertension	283 (58.4)	244 (49.9)
Peripheral vascular disease	8 (1.6)	9 (1.8)
Type of surgery, *n* (%)		
Right colectomy	126 (26.0)	132 (27.0)
Left colectomy	123 (25.4)	124 (25.4)
Total abdominal colectomy	40 (8.2)	37 (7.6)
Low anterior resection	117 (24.0)	122 (25.0)
Other/unspecified	78 (16.1)	74 (15.1)
Abdominal surgery performed, *n* (%)	484 (99.8)	489 (100)
Preoperative IV antibiotics administered, *n* (%)	483 (99.6)	487 (99.6)
Mechanical bowel preparation performed, *n* (%)	406 (83.7)	418 (85.5)
Length of incision (cm)	19.2±6.7	19.5±6.8
Incision length >10 cm to ≤20 cm, *n* (%)	272 (56.2)	278 (56.9)
Incision length >20 cm, *n* (%)	212 (43.8)	211 (43.1)
Anastomosis performed, *n* (%)	426 (88.0)	429 (87.7)
Stoma created, *n* (%)	84 (17.4)	98 (20.0)
Surgery duration (h)	2.7±1.1	2.7±1.1

There were no differences between the groups in any of the above parameters. Data are presented as mean values ± standard deviation or case numbers (%).

CCI, Charlson comorbidity index; COPD, chronic obstructive pulmonary disease; D-PLEX, doxycycline-polymer-lipid encapsulation matrix; SOC, standard of care; SSI, surgical site infection.

a Other benign colorectal diseases: benign neoplasm, diverticulitis coli, ileus (colonic pseudo-obstruction,) volvulus, and intestinal obstruction.

### Efficacy outcomes

#### Primary and secondary outcomes

A total of 104 patients experienced a primary outcome event: 45/485 (9.3%) in D-PLEX versus 59/489 (12.1%) in SOC, but the 23% event reduction in the D-PLEX treatment arm was not significant [risk difference estimate (RDE), −2.8%; 95% CI (−6.7 to 1.0%), *P*=0.1520]. Event rates of individual components were similar in the D-PLEX and SOC arms: Incisional SSI rate (5.8% vs. 6.3%), incisional reinterventions (0 vs. 1.0%), and mortality (2.3% vs. 3.1%) (Table 2). There were no differences between study arms in key secondary outcomes or additional efficacy outcomes, except for the need for any surgical reintervention, for which the rate was reduced in the D-PLEX arm (**eTable 1.**)

**Table 2:** Event rates of the primary outcome, its components, and pre-specified subgroup analysis by incision length (intention-to-treat population).

Overall ITT population			
Outcomes	D-PLEX+SOC (*n*=485)	SOC (*n*=489)	^a^Risk difference estimate, [95% CI], *p*
Primary outcome, *n* (%)	45 (9.3)	59 (12.1)	[−2.8%, (−6.7 to 1.0%), 0.1520]
Incisional SSI events	28 (5.8)	31 (6.3)	
Incisional reintervention events	0	5 (1.0)	
Mortality events	11 (2.3)	15 (3.1)	
Missing day 30 SSI assessment	6 (1.2)	8 (1.6)	
Incision Length (>20 cm) Subgroup			
Outcomes	D-PLEX+SOC (*n*=212)	SOC (*n*=211)	^a^Risk difference estimate, [95% CI], *p*
Primary outcome, *n* (%)	17 (8.0)	37 (17.5)	[−9.4%, (−15.5 to −3.2%), 0.0032]
Incisional SSI events	9 (4.2)	18 (8.5)	
Incisional reintervention events	0	3 (1.4)	
Mortality events	6 (2.8)	10 (4.7)	
Missing day 30 SSI assessment	2 (0.9)	6 (2.8)	
^b^Incision length (>10–≤20 cm) subgroup			
Outcomes	D-PLEX+SOC (*n*=272)	SOC (*n*=278)	^a^Risk difference estimate, [95% CI], *p*
Primary outcome, *n* (%)	27 (9.9)	22 (7.9)	[2.0%, (−2.8 to 6.7%), 0.4133]
Incisional SSI events	19 (7.0)	13 (4.7)	
Incisional reintervention events	0	2 (0.7)	
Mortality events	5 (1.8)	5 (1.8)	
Missing day 30 SSI assessment	3 (1.1)	2 (0.7)	

D-PLEX, doxycycline-polymer-lipid encapsulation matrix; ITT, intention-to-treat; SOC, standard of care; SSI, surgical site infection.

a Stratified risk difference estimate.

b One patient was excluded from the analysis because the incisional length was not available. The primary outcome was defined per protocol at the patient level and included only one of the following within 30 days post-abdominal index operation: Incisional SSI event (superficial sSSI or deep dSSI), incisional reintervention in the index surgical incision due to poor wound healing, including wound dehiscence, or mortality (for any reason). Patients with missing primary efficacy outcome data for reasons other than death were considered as failures. Patients experiencing more than one type of event were counted only once, in the highest category, based on the following order: Death, SSI event, incisional reintervention, and missing day 30 SSI assessment.

#### Pre-specified subgroup analyses

In the greater than 20 cm incision length subgroup, a 54% risk reduction of the primary outcome was observed in D-PLEX (8%, 17/212) compared with SOC (17.5%, 37/211) [RDE, −9.4%; 95% CI (−15.5 to −3.2%); *P*=0.0032]. However, in the greater than 10 to less than or equal to 20 cm incision length subgroup, no reduction was observed: 9.9% (27/272) in D-PLEX versus 7.9% (22/278) in SOC [RDE, 2.0%; 95% CI (−2.8 to 6.7%), *P*=0.4133] (Table 2). Regarding the analysis of the primary outcome incidence by preoperative MBP, there was no difference in terms of D-PLEX effect with or without preoperative MBP (eTable 2, Supplement 1, Supplemental Digital Content 1, http://links.lww.com/JS9/C735).

Analysis of the key secondary efficacy outcome, incisional SSI, also indicated a 54.6% risk reduction in the greater than 20 cm incision subgroup (4.4% in D-PLEX vs. 9.7% in SOC, *P*=0.0410); however, no reduction was observed in the greater than 10 to less than or equal to 20 cm incision length subgroup (7.5% in D-PLEX vs. 4.8% in SOC, *P*=0.2014) (Table 3). The limited number of patients with ASEPSIS scores greater than 20 precluded the analysis of the effect of incision length on this key secondary outcome (Table 3).

**Table 3:** Secondary efficacy outcomes at 30 days post-operation by incision length.

Parameter	Incision >10–≤20 cm			Incision >20 cm		
	D-PLEX+SOC *N*=272	SOC *N*=278	*P*	D-PLEX+SOC *N*=212	SOC *N*=211	*P*
Key secondary outcomes: pre-specified analysis, *n* (%)						
Incisional SSI rate	20/265 (7.5)	13/271 (4.8)	0.2014	9/204 (4.4)	19/196 (9.7)	0.0410
At least 1 score of ASEPSIS >20	6/265 (2.3)	5/271 (1.8)	0.7241	2/204 (1.0)	5/196 (2.6)	0.2231
Additional efficacy secondary outcomes: post hoc analysis						
sSSI rate, *n* (%)	17/264 (6.4)	12/271 (4.4)	0.3393	9/204 (4.4)	17/196 (8.7)	0.0899
dSSI rate, *n* (%)	3/265 (1.1)	1/271 (0.4)	0.3681	0/204 (0)	2/194 (1.0)	0.237
All-cause mortality rate, *n* (%)	5 (1.8)	5 (1.8)	0.9703	6 (2.8)	10 (4.7)	0.2939
Time to SSI (day)	11.0	8.0	0.5165	8.0	5.0	0.0819
Surgical reintervention-any cause, *n* (%)	10/267 (3.7)	15/272 (5.5)	0.3299	9/206 (4.4)	19/196 (9.7)	0.0333
Incisional reintervention, *n* (%)	3/265 (1.1)	3/271 (1.1)	1.000	0/204 (0)	4/194 (2.1)	0.0556

*P* values were calculated as follows: all-cause 30-day mortality rates were based on the Z test, and days to SSI were based on the Wilcoxon rank-sum test. The rest of the parameters (categorical Yes/No variables) were based on either the Cochran–Mantel–Haenszel test or Fisher exact test.

D-PLEX, doxycycline-Polymer-Lipid Encapsulation matriX; dSSI, deep incisional surgical site infection; SOC, standard of care; SSI, surgical site infection; sSSI, superficial incisional surgical site infection.

#### Post hoc analyses

Exploratory analysis of the additional secondary efficacy outcomes indicated marked differences in favor of D-PLEX vs. SOC in the greater than 20 cm incision length subgroup. Notably, the need for any surgical reintervention decreased by 54.6% in the D-PLEX treatment arm compared to the SOC (4.4% vs. 9.7%, *P*=0.0333). In the greater than 10 to less than or equal to 20 cm incision length subgroup, there were no significant differences in terms of additional secondary efficacy outcomes between the study arms (Table 3).

In exploratory analyses of subjects with at least one patient-specific risk factor, the primary outcome occurred in 9% (31/345) of the patients in the D-PLEX arm and 13.7% (46/335) in the SOC arm [RDE, −4.8%; 95% CI (−9.5% to −0.1%), *P*=0.0472]. In patients with ≥1 patient-specific risk factor or procedural factor, the primary outcome event occurred in 8.6% (35/408) of the patients in the D-PLEX arm and 13.7% (56/408) in the SOC arm [RDE, −5.3%; 95% CI (−9.5 to −1.0%), *P*=0.0162] (Table 4).

**Table 4:** The primary outcome results in patients with SSI risk factors: post hoc analyses

SSI Risk	D-PLEX+SOC	SOC	^a^Risk difference estimate, [95% CI], *P*
^b^≥1 patient-specific risks, *n* (%)	31/345 (9.0)	46/335 (13.7)	[−4.8%, (−9.5 to −0.1%), 0.0472]
^b^≥1 patient-specific or ^c^procedural risks, *n* (%)	35/408 (8.6)	56/408 (13.7)	[−5.3%, (−9.5 to −1.0%), 0.0162]

D-PLEX, doxycycline-polymer-lipid encapsulation matrix; SOC, standard of care; SSI, surgical site infection.

a Stratified risk difference estimates.

b Patient-specific risk factors assessed: obesity/overweight (BMI ≥30 kg/m^2^), diabetes mellitus, hypertension, peripheral vascular disease, and chronic obstructive pulmonary disease/reported smoking history.

c Procedural risk factor: patients undergoing surgical incision >20 cm Analysis statistics were obtained from the Cochran–Mantel–Haenszel test using the study stratification factors used at randomization. Stratified risk differences with a 95% CI were estimated using the Mantel–Haenszel stratum weights method.

In exploratory analyses of the 772 subjects who underwent colorectal cancer resection, the primary outcome occurred in 8.9% (34/382) of the patients in the D-PLEX arm and 12.1% (47/390) in the SOC arm [RDE, −3.3%; 95% CI (−7.6 to −1%), *P*=0.1337].

#### Safety

The study safety population included 976 patients: 478 with D-PLEX and 498 with SOC. The overall incidence of treatment-emergent adverse events (TEAEs) was similar between the study arms, with numerically lower incidences of severe and serious TEAEs, and any TEAEs requiring surgical reinterventions in the D-PLEX arm compared to the SOC arm. (eTable 3., Supplement 1. Supplemental Digital Content 1, http://links.lww.com/JS9/C735)

## Discussion

This large multinational double-blind randomized clinical trial assessed the efficacy and safety of locally administered D-PLEX for the prevention of incisional SSIs in patients undergoing abdominal colorectal surgery. Although D-PLEX produced a numerical reduction in the incidence of the primary outcome events, it did not reach statistical significance.

This study faced multiple challenges because it was conducted during the height of the COVID-19 pandemic^42^, when overall hygiene awareness was intensified in hospitals and lower SSI rates were reported compared to the pre-pandemic period^43–46^. Consequently, a lower-than-expected incisional SSI rate was observed in the control arm versus the initially assumed baseline rate (6.9% rather than 16.0%), which reduced the statistical power and thus the potential to demonstrate a significant effect with D-PLEX treatment. This was demonstrated in the pre-specified analysis of the key secondary outcomes by incision length: In the greater than 20 cm incision length subgroup, where a relatively high incisional SSI event rate was observed in the control arm (9.7%), D-PLEX provided a 54% reduction of the incisional SSI rate (4.4%, *P*=0.0410). This effect, in turn, yielded a meaningful reduction in the primary outcome events (54%, *P*=0.0032) in this subgroup. In the greater than 10 to less than or equal to 20 cm incision length subgroup, the incisional SSI event rate was markedly lower in the control arm (4.8%), and no D-PLEX effect was observed, possibly because such a low event rate would be difficult to reduce further.

Surgical incision length is a well-known independent risk factor for SSI in abdominal operations^32–36^. In the subgroup of patients with procedural risk undergoing surgery (>20 cm incision length; 43% of the ITT population), we observed not only a significant reduction in the incidence of the primary outcome but also reductions in several secondary efficacy endpoints, showing a consistent effect.

Additional exploratory post hoc analyses of patients with patient-specific SSI risk factors, in whom a higher rate of incisional SSI was expected, were confirmatory. Among the subjects with greater than or equal to 1 patient-specific risk (70% of the ITT population), a 34% rate reduction was observed in the composite primary outcome (*P*=0.0472). This result is consistent with our Phase 2 data in this population, which demonstrated incisional SSI reduction in subjects with patient-specific risks^41^. Additional post hoc analyses of subjects with ≥1 patient-specific risk factor or procedural risk factor (84% of the ITT population) also demonstrated a reduction in the primary outcome with D-PLEX (37% reduction, *P*=0.0162). Together, these results suggest potential prophylactic efficacy of D-PLEX when administered concomitantly with systemic antibacterial prophylaxis in patients with increased SSI risk factors, whether procedural or patient-specific comorbidities. Patients with either of these risk profiles are readily identifiable by the surgeon in the preoperative and intraoperative periods, offering the option of applying D-PLEX after fascial closure but before skin closure. Moreover, the present study demonstrated an overall good safety profile for D-PLEX, confirming the safety profile previously observed in the D-PLEX phase 2 study^24^.

The possible benefit of D-PLEX in the clean-contaminated wound environment may be due to its effective local prolonged-release mechanism, providing a high local concentration of doxycycline for 30 days that exceeds the MIC of doxycycline for most common SSI pathogens^23^. Several topical antimicrobial agents (both antibiotics and antiseptics) have been investigated previously to complement or supplement systemic antibacterial prophylaxis; however, most demonstrated questionable results or low-quality evidence^47–49^. In particular, studies evaluating topical antibiotics in abdominal colorectal surgery procedures demonstrate a lack of efficacy for reduced SSI rates^50–53^.

### Limitations

Our study had several limitations. Conducting the study during the peak of the COVID-19 pandemic caused an unexpected decrease in SSI control rates compared to the pre-pandemic period, which may have negatively impacted the potential to demonstrate a statistically significant effect of D-PLEX treatment^43–46^. Although the trial was conducted in 10 different countries, it consisted predominantly of a Caucasian Eastern European population, which may limit the generalizability of the results. In addition, the pre-specified sensitivity analysis by incision length was used as a stand-alone analysis without compensation for multiplicity. The fact that MBP was left to the discretion of the operating surgeon is a potential limitation, although the groups were balanced^54,55^. Finally, D-PLEX was assessed in a setting that excluded OABP; therefore, concurrent administration could not be assessed. These limitations are currently being addressed in a second phase 3 trial (SHIELD 2, ClinicalTrials.gov identifier; NCT04411199).

## Conclusions

The SHIELD 1 study did not meet its primary composite outcome of incisional SSI, incisional reinterventions, or all-cause mortality. However, in a pre-specified analysis of the subpopulation with lengthy surgical incisions (>20 cm), there was a clinically meaningful reduction in the primary outcome events. This effect was corroborated by the results of the secondary outcomes. Exploratory post hoc analyses of patients with increased SSI risk (procedural or patient-specific SSI risk) also demonstrated a substantial reduction in the primary outcome events after D-PLEX use, supporting the potential prophylactic use of D-PLEX in patients with identified SSI risk factors at the time of abdominal colorectal surgery. D-PLEX demonstrated an overall good safety profile. D-PLEX efficacy and safety among patients with increased SSI risk is currently being evaluated in an additional Phase 3 clinical trial (SHIELD 2).

## Ethical approval

1. Oded Zmora—Helsinki committee, Shamir Medical Center, Israel, approval # 0028-20-ASF
2. Lior Segev—Helsinki committee, The Chaim Sheba Medical Center, Israel, approval # 7947-20-SMC
3. Philip Fleshner—Office of Research Compliance and Quality Improvement, Cedars-Sinai MC, US, IRB protocol ID # STUDY00000964.

## Consent

All patients signed an informed consent form prior to their study entry.

## Source of funding

The study was designed and sponsored by PolyPid Ltd. Both authors and sponsor (PolyPid) were involved in in the collection, analysis and interpretation of data; in the writing of the manuscript; and in the decision to submit the manuscript for publication. This research did not receive any public or not-for-profit sector funds.

## Author contribution

O.Z.: investigation and writing (review and editing). P.F.: investigation and writing (review and editing). P.S.B.: visualization and writing (review and editing). L.S.: investigation and writing (review and editing). G.M.V.: investigation (primary endpoint adjudication) and writing (review and editing). A.J.S.: validation and writing (review and editing). A.S.: investigation (primary endpoint adjudication) and writing (review and editing). O.B.: conceptualization, validation, visualization and writing (review and editing). S.S.: validation and writing (review and editing). N.E.: conceptualization, supervision and writing (review and editing).

## Conflicts of interest disclosure

P.S.B. was a consultant to the sponsoring company as part of a scientific advisory board held on the study results. G.M.V. and A.S. were members of the adjudication committee. N.E., O.B., A.J.S. and S.S. are paid employees or scientific advisors of the sponsoring company and own stock/stock options. No other disclosures were reported.

## Research registration unique identifying number (UIN)

Our clinical trial was registered in https://www.clinicaltrials.gov/ publicly accessible database before recruitment of the first subject under assigned Unique Identifying Number (UIN): NCT04233424.

## Guarantor

Noam Emanuel.

## Data availability statement

Data sharing is not applicable to this article as data will serve as a basis for a regulatory filing and cannot be shared at this time.

## Provenance and peer review

The present paper was not invited.

## Data sharing statement

Data will serve as a basis for a regulatory filing and cannot be shared at this time.

## Supplementary Material

SUPPLEMENTARY MATERIAL
