# Supplementary material for: Effect of local prolonged-release incisional doxycycline on surgical site infection prophylaxis in abdominal colorectal surgery: the SHIELD 1 randomized clinical trial
Source: Int J Surg. 2024 Jun 13;110(10):6658–66. doi: 10.1097/JS9.0000000000001824 (PMC11486998; doi:10.1097/JS9.0000000000001824)
Supplement: SUPPLEMENTARY MATERIAL [file js9-110-6658-s001.docx]

**Supplementary Online Content: Supplement 1**

Barie et al. Effect of A Local Prolonged-Release Incisional Doxycycline on Surgical Site Infection Prophylaxis in Abdominal Colorectal Surgery: The SHIELD 1 Randomized Clinical Trial

**eFigure 1.** D-PLEX: Preparation and Administration

**eTable 1.** Secondary Efficacy Outcomes at 30 Days Post-operation (ITT Population)

**eTable 2.** Primary Outcome Results by Preoperative MBP

**eTable 3.** Treatment-Emergent Adverse Events (TEAE) - Safety Population

This supplementary material is provided by the authors to provide the readership with additional information on their work.

**eFigure 1. D-PLEX: Preparation and Administration**


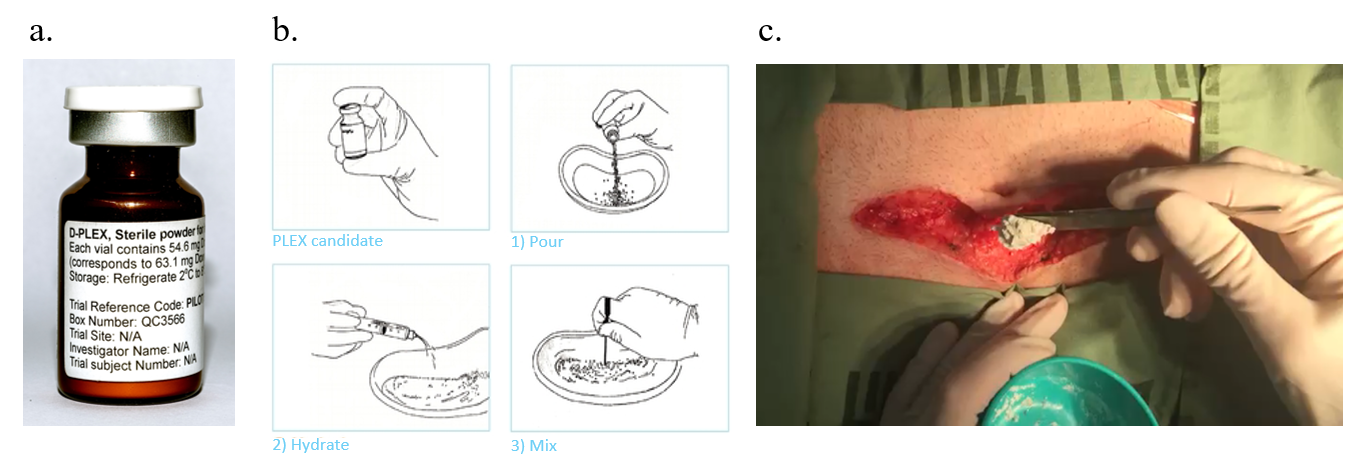


a. D-PLEX vial, b. D-PLEX reconstitution, and c. D-PLEX is being applied to the incision edges at closure. D-PLEX: Doxycycline-Polymer-Lipid Encapsulation matriX.

**eTable 1. Secondary Efficacy Outcomes at 30 Days Post-operation (ITT Population)**

|  | D-PLEX+SOC (n=485) | SOC (n=489) | ^a^ Risk Difference Estimate, [95% CI], p |
| --- | --- | --- | --- |
| **Key secondary outcomes** |  |  |  |
| SSI Events | 29/469 (6.2%) | 32/467 (6.9%) | (-0.8%, [-3.9% to 2.3%], 0.6219) |
| ASEPSIS >20 (Events) | 8/469 (1.7%) | 10/467 (2.1%) | (-0.4%, [-2.2% to 1.3%], 0.6328) |
| **Additional secondary efficacy outcomes** | | | |
| sSSI rate | 26/468 (5.6%) | 29/467 (6.2%) | (-0.8%, [-3.8% to 2.2%], 0.6111) |
| dSSI rate | 3/469 (0.6%) | 3/465 (0.6%) | (-0.01%, [-1.03% to 1.02%], 1.0000) |
| All-cause mortality rate | 11/485 (2.3%) | 15/489 (3.1%) | (-0.8%, [-2.8% to 1.2%], 0.4373) |
| Time to SSI [days, (range)] | 11.0 (4, 30) | 7.0 (1, 25) | 0.0196 |
| Surgical reintervention-any cause | 19/473 (4.0%) | 34/468 (7.3%) | (-3.3%, [-6.2% to 0.3%], 0.0292) |
| Incisional reintervention | 3/469 (0.6%) | 7/465 (1.5%) | (-0.9%, [-2.25 to 0.5%], 0.2223) |

Abbreviations: SSI, surgical site infection; ITT, Intention-to-treat; SOC, standard of care; D-PLEX, Doxycycline-Polymer-Lipid Encapsulation matriX; ASEPSIS, Additional treatment, the presence of Serous discharge, Erythema, or Purulent exudate score; Separation of the deep tissues, Isolation of bacteria, and duration of inpatient Stay; sSSI, superficial incisional surgical site infection; dSSI, deep incisional surgical site infection. Incisional reintervention, reintervention in the index surgical incision due to poor wound healing including wound dehiscence. P values were calculated as follows: All-cause 30-day mortality rates were based on the Z test, and days to SSI were based on the Wilcoxon Rank-Sum test. The rest of the parameters (categorical Yes/No variables) were based on either Cochran–Mantel–Haenszel test or the Fisher exact test. ^a^Stratified risk difference estimate. Stratified risk differences with a 95% confidence interval were estimated using the method of Mantel–Haenszel stratum weights.

**eTable 2.** Primary Outcome Results by Preoperative MBP

| **Preoperative MBP** | D-PLEX+SOC | SOC | ^a^ Risk Difference Estimate, [95% CI], p |
| --- | --- | --- | --- |
| With MBP | 31/406 (7.6%) | 44/418 (10.5%) | (-2.9%, [-6.8% to 1.0%], 0.1825) |
| Without MPB | 14/79 (17.7%) | 15/71 (21.1%) | (-3.4%, [-16.1% to 9.3%], 0.6805) |

Abbreviations: MBP, mechanical bowel preparation; SSI, surgical site infection; SOC, standard of care; D-PLEX, doxycycline-Polymer-Lipid Encapsulation matriX. ^a^Stratified risk difference estimate. Analysis statistics were obtained from the Cochran–Mantel–Haenszel test using study stratification factors used at randomization. Stratified risk differences with a 95% confidence interval were estimated using the method of Mantel–Haenszel stratum weights.

**eTable 3.** Treatment-Emergent Adverse Events (TEAE)-Safety Population

|  | **D-PLEX+SOC (N=478)** | | **SOC (N=498)** | |
| --- | --- | --- | --- | --- |
| **Category** | **n (%)** | **No. of Events** | **n (%)** | **No. of Events** |
| Patients with at least one TEAE | 381 (79.7) | 1,301 | 398 (79.9) | 1,448 |
| Patients with at least one severe TEAE | 58 (12.1) | 89 | 81 (16.3) | 128 |
| \| Patients with at least one serious TEAE \| 69 (14.4) \| 114 \| 98 (19.7) \| 155 \| \| --- \| --- \| --- \| --- \| --- \| \| Patients with any TEAE resulting in any surgical reintervention \| 26 (5.4) \| 40 \| 45 (9.0) \| 62 \| \| Patients with any TEAE resulting in surgical reintervention of the index incision \| 20 (4.2) \| 34 \| 35 (7.0) \| 49 \| \| Patients with any TEAE resulting in death \| 16 (3.3) \| 25 \| 17 (3.4) \| 24 \| | | | | |
| Patients with any investigational product-related TEAE | 28 (5.9) | 39 | 24 (4.8) | 29 |

Abbreviation: SOC, Standard of care; D-PLEX, Doxycycline-Polymer-Lipid Encapsulation matriX. TEAEs are defined as all adverse events that start on or after the date of the index operation.
